# Supplementary material for: Systematic mapping of contact sites reveals tethers and a function for the peroxisome-mitochondria contact
Source: Nat Commun. 2018 May 2;9:1761. doi: 10.1038/s41467-018-03957-8 (PMC5932058; doi:10.1038/s41467-018-03957-8)
Supplement: Supplementary file 1 — Supplementary Information [file 41467_2018_3957_MOESM1_ESM.pdf]

Systematic mapping of contact sites reveals tethers and a function for the peroxisome-mitochondria contact

Shai *et al.*

Supplementary information

Shai *et al*. Supplementary Figure 1

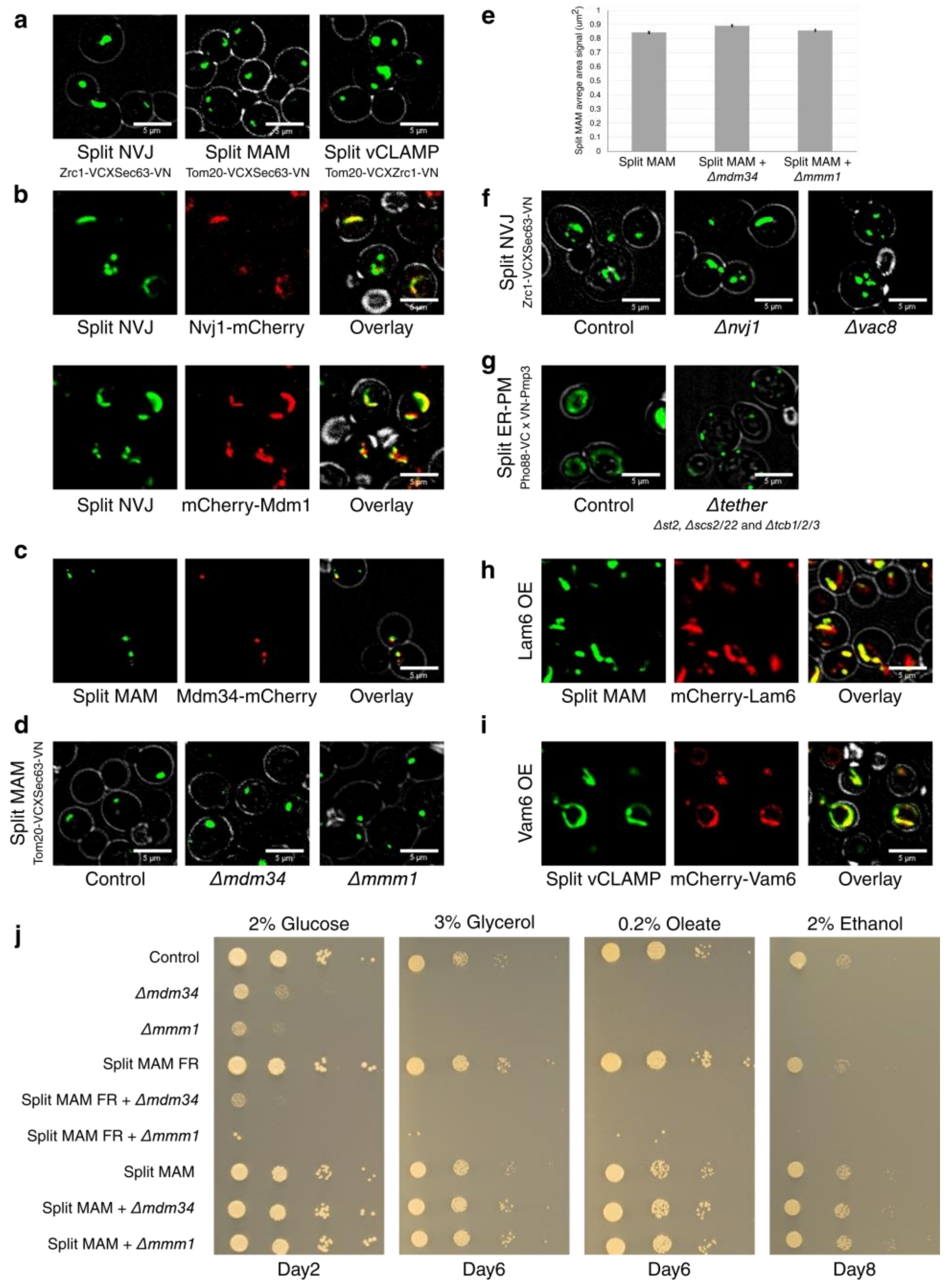

Supplementary Figure 1. Split Venus reporters can be used to systematically study contact sites. a We created split Venus reporters for the three most studied contact sites in yeast: Vacuole-ER (NVJ), mitochondria-ER (MAM) and vacuole–mitochondria (vCLAMP). **b, c** Known contact site resident proteins co-localize with their respective contact site reporter: Nvj1 and Mdm1 to the NVJ reporter (**b**) and Mdm34 to the MAM reporter (**c**). Thus the fluorescent signal represents the actual contact site. **d, e, f** Single deletions of known tethers do not affect the split reporter signal: The MAM reporter signal was not changed when known MAM tether genes, *MDM*34 or *MMM1*, were deleted (**d**). Deleting *MDM34* or *MMM1* did not affect the average MAM reporter signal

(um^2). Bar represent mean±s.e. from three independent experiments (**e**). The NVJ reporter signal was not changed when the NVJ tether genes, *NVJ1* or *VAC8*, were deleted (**f**). **g** The PM-ER reporter contact site was severely affected by deletions of six known tethering genes (Δ*tether)*. **h, i** The split Venus reporters are affected by manipulating known contact site tethers and regulators: The MAM reporter signal was expanded when Lam6, a known tether of the MAM, was overexpressed (**h**). The vCLAMP reporter signal was expanded when Vam6, a known tether of the vCLAMP, was overexpressed (**i**). **j** A serial dilution growth assay was carried out for control or *Δermes (Δmdm34/Δmmm1*) cells, with or without the MAM reporters (Venus or FR) on agar plates containing either 2% glucose, 3% glycerol, 0.2% oleate or 2% ethanol. The MAM reporter, but not the MAM Far Red (FR) reporter, rescued the ability of the *Δermes* strains to grow on glycerol, oleate and ethanol.

Shai *et al*. Supplementary Figure 2

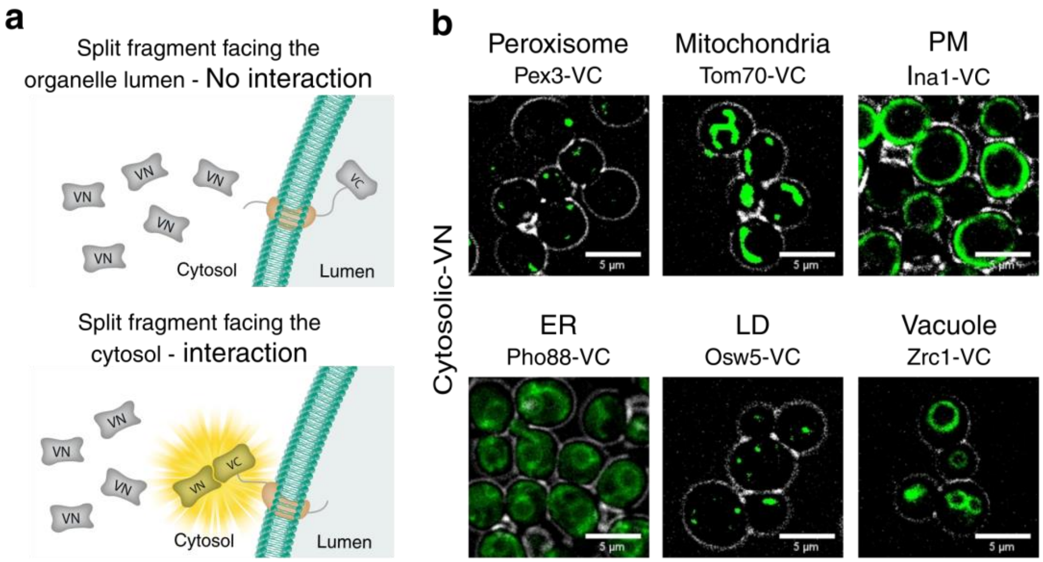

Supplementary Figure 2. Optimizing the split Venus reporters.

a Verifying the localization, expression and orientation of the tagged membrane proteins. To ensure that the candidate proteins are expressed, localized to the correct organelle and that their tagged terminus is facing the cytosol, all candidate proteins were tagged at their C terminus with half of the Venus protein (VC), while the other half (VN) was expressed in the cytosol. **b** Representative images of different membrane proteins that were localized to the desired organelle leading to a fluorescent pattern that is typical to the organelle.

Shai *et al*. Supplementary Figure 3

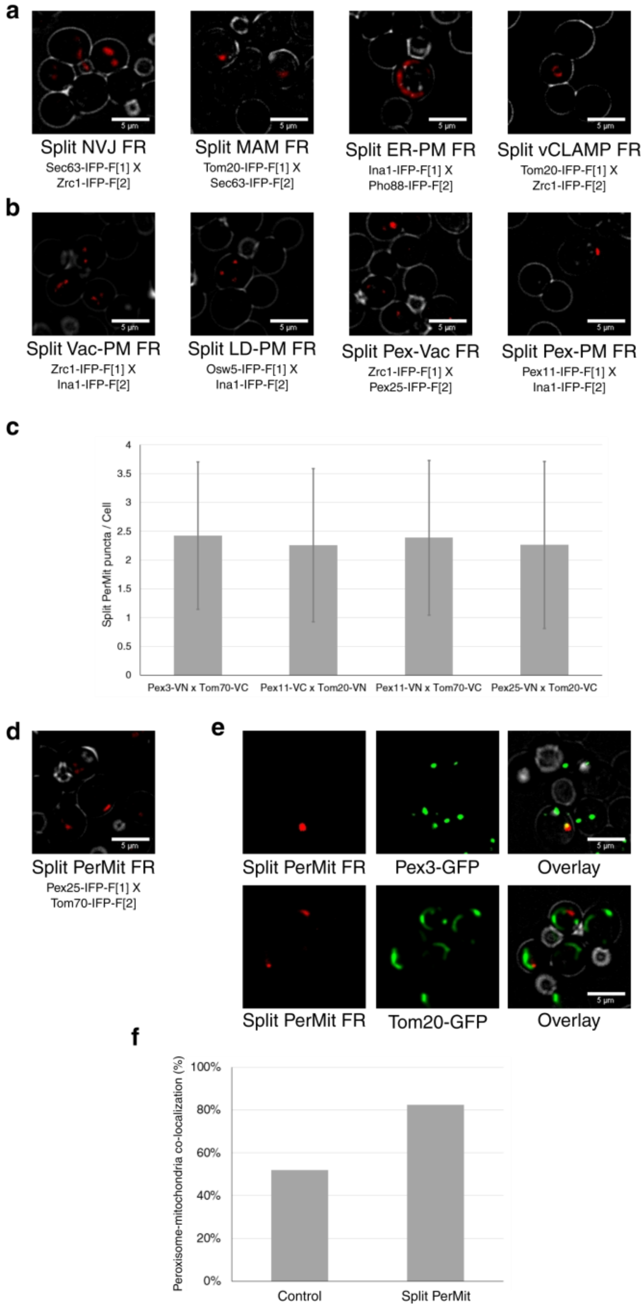

Supplementary Figure 3. Assaying the capacity of the split reporters to form synthetic tethers. a Split Far Red (FR) reporters were made for known contact sites NVJ, MAM, ER-PM and vCLAMP. **b** Split FR reporters show contacts for the four new cell compartment proximities discovered using split Venus: vacuole-PM (vCOuPLE), LD-PM (pCLIP), peroxisome-vacuole (PerVale) and peroxisome-PM (PerPECs). **c** Quantification of PerMit puncta number per cell for strains expressing different split PerMit reporters. The results show that there was no difference in PerMit puncta when the different reporters were used. Bar represent mean±s.d. from three

independent experiments (at least 600 cells from each strain). **d** Peroxisome-mitochondria proximities can be visualized using a split FR reporter. **e** The PerMit FR reporter signal co-localizes to both a peroxisomal marker (Pex3-GFP) and to a mitochondrial marker (Tom20-GFP) demonstrating that it indeed marks sites of close apposition between the two membranes. **f** Quantification of peroxisomes that co-localized with mitochondria with or without the split Venus PerMit reporter. The results imply that the split Venus PerMit reporter stabilizes organelle contacts. Bar represents mean.

Shai *et al*. Supplementary Figure 4

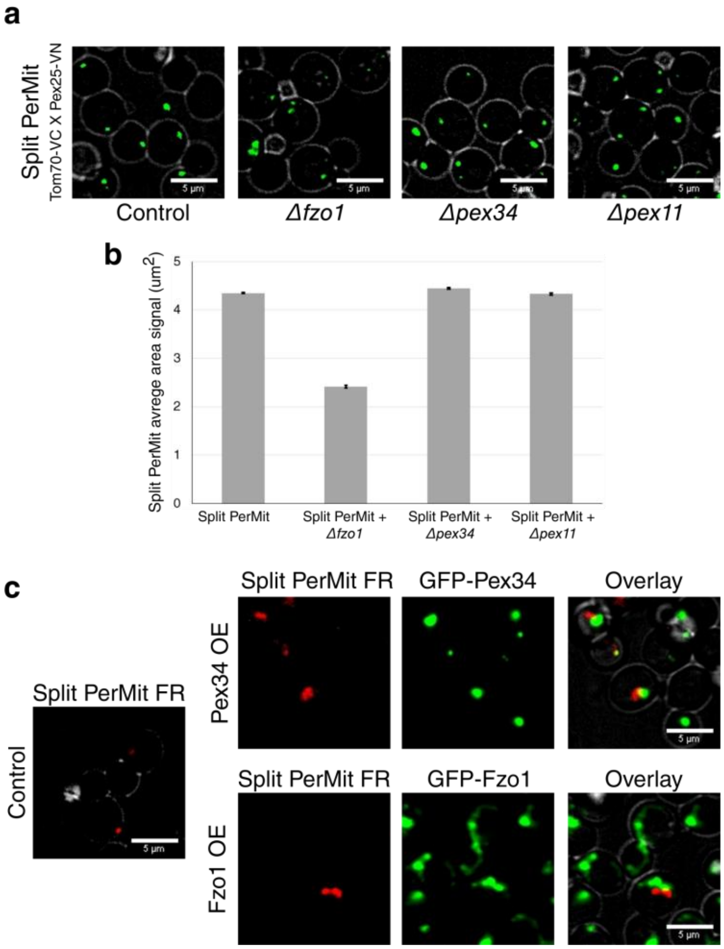

Supplementary Figure 4. Fzo1 and Pex34 have the characteristics of potential PerMit tethers. a Deletions of *PEX34*, the newly suggested PerMit tether, or *PEX11*, a previously suggested PerMit tether, did not affect the PerMit reporter signal. Deletion of *FZO1*, known to affect mitochondria morphology, affected the PerMit signal but this might be caused by altering mitochondria shape. **b** Quantification of the average PerMit reporter signal (um^2) in control cells or in the background of *FZO1*, *PEX34* or, *PEX11* deletions. Bar represent mean±s.e from three independent experiments. The results demonstrate that single deletions of *PEX34* or *PEX11* do not affect the PerMit signal. **c** Pex34 or Fzo1 overexpression (Tef2-GFP-Pex34/Fzo1) led to expansion of the PerMit FR reporter demonstrating that the PerMit expansion is not specific to the presence of the split Venus reporter.

Shai *et al*. Supplementary Figure 5

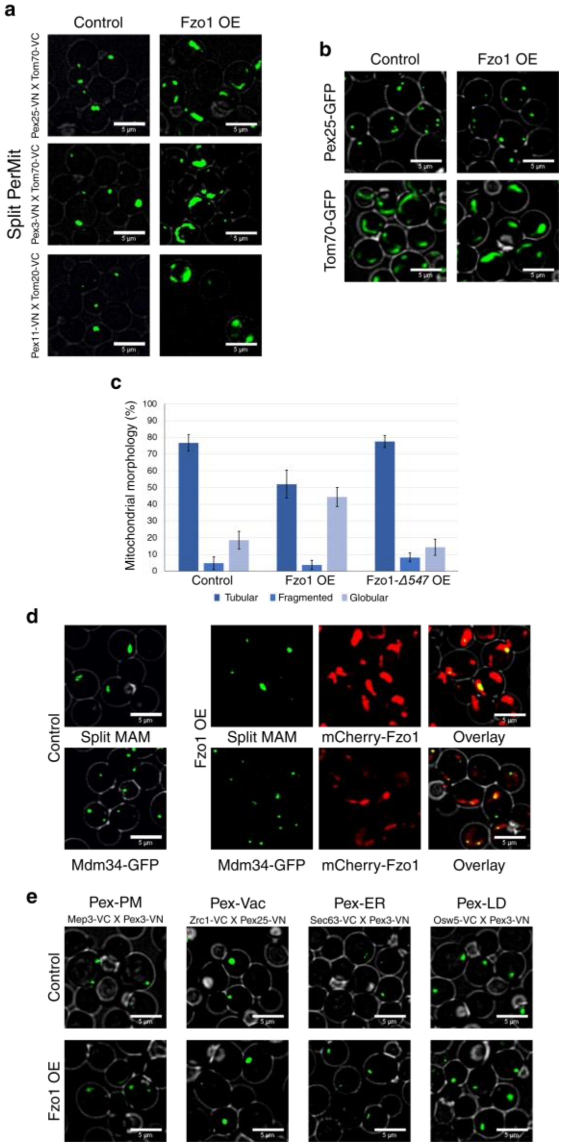

Supplementary Figure 5. Fzo1 overexpression specifically expands the PerMit contact site. a Fzo1 overexpression (OE) expands different PerMit reporters regardless of the tagged proteins used. **b** Fzo1 OE led to hyper-fusion of mitochondria, detected by Tom70. However, the overexpression did not affect the expression of Pex25. **c** Quantification of mitochondrial morphology assessed by following Tom70-GFP fluorescence in control, Fzo1 OE or truncated Fzo1(*Δ547*) OE cells. Error bars represent the s.d. from three independent experiments. The results show that the PerMit expansion observed when truncated Fzo1 is overexpressed is not a result of altering mitochondria morphology. **d** Fzo1 OE does not affect the MAM reporter nor the native ERMES complex (marked using Mdm34-GFP) implying a direct effect on the PerMit contact. **e** Fzo1 OE does not affect any of the other peroxisomal contact site reporters suggesting a specific tethering role in the PerMit contact.

Shai *et al*. Supplementary Figure 6

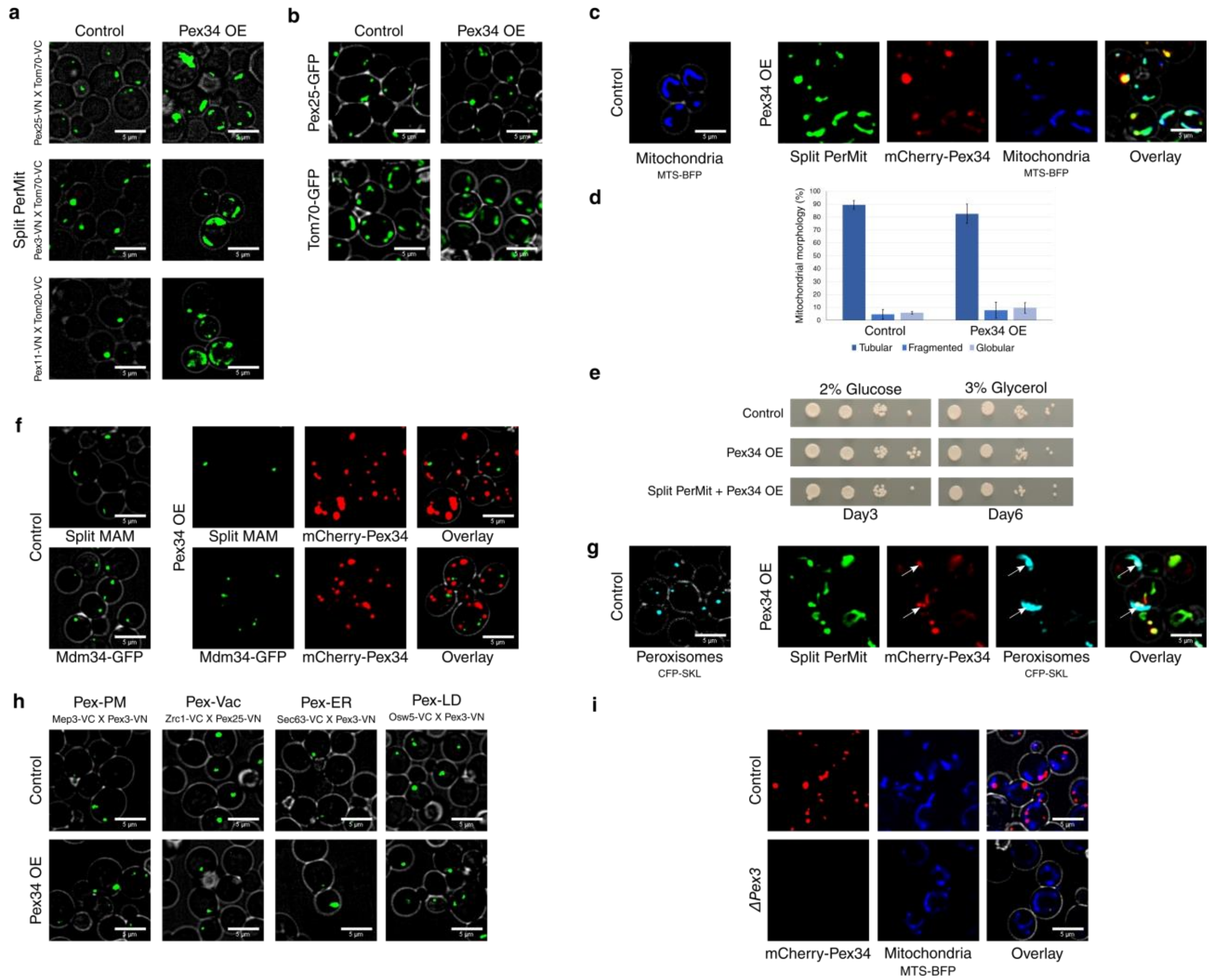

Supplementary Figure 6. Pex34 overexpression specifically expands the PerMit contact site. **a** Pex34 Overexpression (OE) expands different PerMit reporter signals regardless of the tagged protein used. **b** Pex34 OE does not affect the expression or localization of Pex25 and Tom70 demonstrating that the PerMit expansion is not a result of indirect effects on the tagged proteins. **c** Pex34 OE does not affect mitochondrial structure (marked by MTS-BFP). **d** Quantification of mitochondrial morphology assessed by following MTS-BFP fluorescence in control, or Pex34 OE cells. Error bars represent the s.d. from three independent experiments. The results imply that the PerMit expansion observed when Pex34 is overexpressed is not a result of altering mitochondria morphology. **e** A serial dilution growth assay was carried out for control or Pex34 overexpression cells, with or without the split PerMit reporter on agar plates containing either 2% glucose or 3% glycerol. Pex34 overexpression strains are able to grow on glycerol implying that Pex34 OE does not affect the ability to respire. **f** Pex34 OE does not affect the MAM reporter nor the ERMES complex (marked using Mdm34-GFP) implying a direct effect of Pex34 on the PerMit signal. **g** Pex34 OE leads to peroxisome (marked by CFP-SKL) clumping on mitochondria. Pex34 is

enriched in specific peroxisomal niches (White arrows). **h** Pex34 OE does not affect any of the other peroxisomal contact site reporters suggesting a specific role in tethering the PerMit contact. **i** Pex34 is not targeted to mitochondria even when it is overexpressed and no mature peroxisomes exist (*Δpex3*). This demonstrates that the PerMit expansion is not caused by mistargeting of Pex34 to mitochondria.

Shai *et al*. Supplementary Figure 7

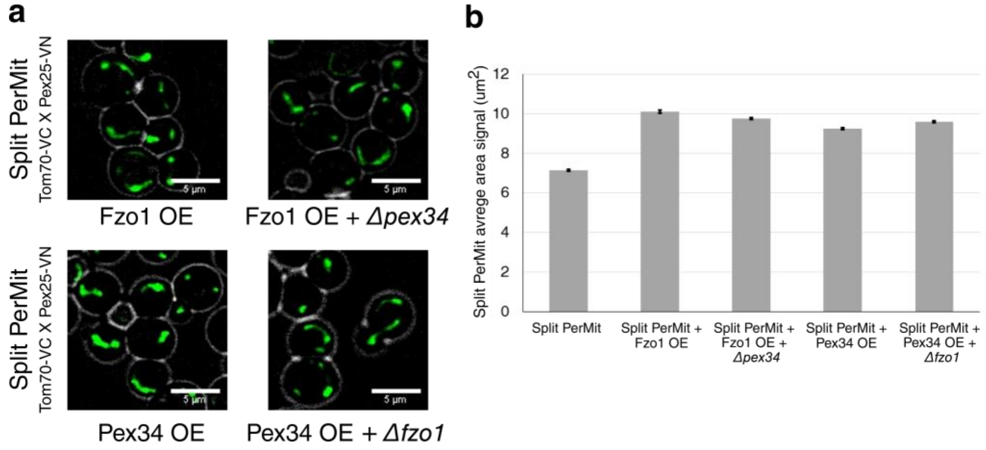

Supplementary Figure 7. Pex34 and Fzo1 function as different PerMit tether complexes. a Deletion of *PEX*34 does not affect the ability of Fzo1 overexpression to expand the PerMit contact and *vice versa*. **b** Quantification of the average PerMit reporter signal (um^2). Bar represent mean±s.e. from three independent experiments. The results imply that Pex34 and Fzo1 are not part of the same PerMit tethering complex.

Shai *et al*. Supplementary Figure 8

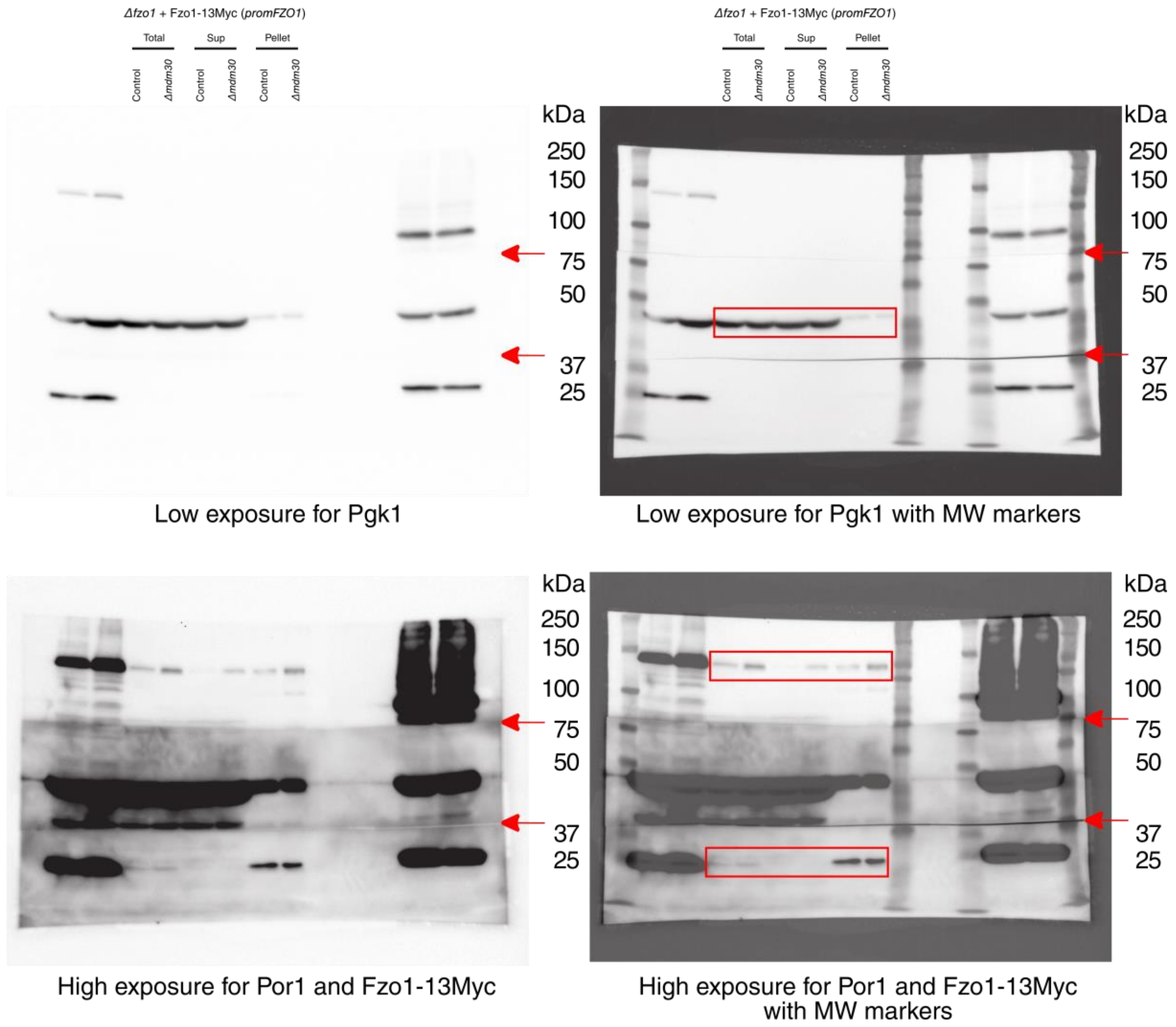

Supplementary Figure 8. Whole scans of blots shown in Figure 4c. Low exposure for Pgk1 without (left) and with (right) molecular weights markers are shown in the upper panels. High exposure for Por1 and Fzo1-13Myc without (left) and with (right) molecular weights markers are shown in the lower panels. Molecular weights indicated in kDa. Red boxes mark the parts of the Western blot images that are shown in the indicated figure. Red arrows mark membrane cuts for different primary antibody incubation.

Shai *et al*. Supplementary Figure 9

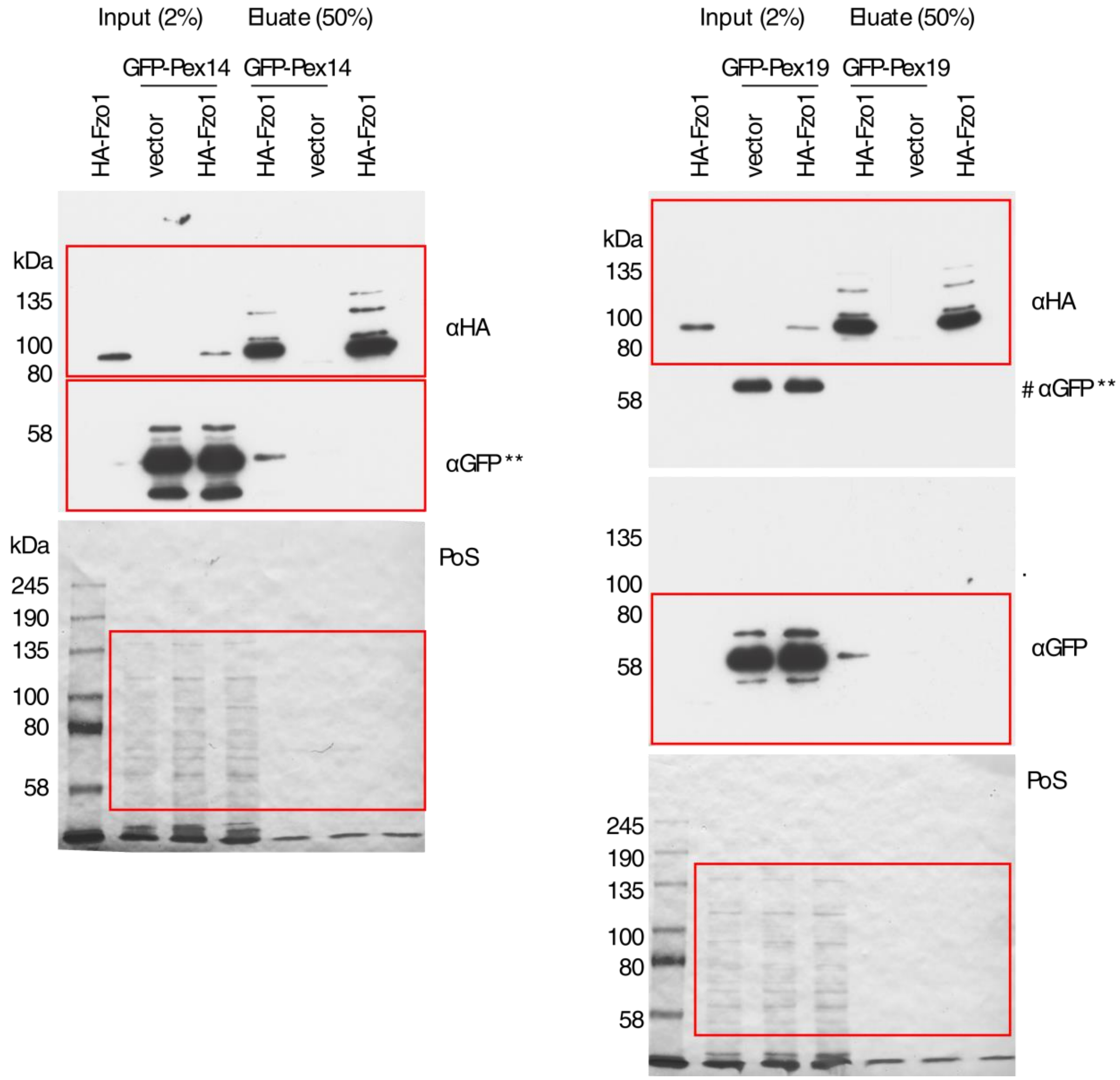

Supplementary Figure 9. Whole scans of blots shown in Figure 4f. αGFP** - anti-GFP immunoblotting performed after previous anti-HA immunoblotting of the same membrane, hence explaining the signals of both antibodies on the same blot. # - denotes a panel not shown in Figure 4f. Ponceau S staining (PoS) was used to compare protein levels. Molecular weights indicated in kDa. Red boxes mark the parts of the Western blot images that are shown in the indicated figure.

Supplementary Table 1 – The membrane proteins from each cell compartment that were utilized for reporter formation.

Cell compartment	Protein	Abundance (Molecules per cell)	Localization	Orientation of C'	Detected with cytoplasmic split VN?
**Peroxisome**	Ant1	2250	Peroxisome	Cytoplasm	X
	Pex3	1400	Peroxisome	Cytoplasm	√
	Pex11	1630	Peroxisome	Unknown	√
	Pex25	2420	Peroxisome	Cytoplasm	√
	Pex30	4570	Peroxisome	Cytoplasm	√
**Mitochondria**	Mdm10	768	Mitochondria	Cytoplasm	√
	Mdm34	377	Mitochondria	Cytoplasm	X
	Tom20	5680	Mitochondria	Cytoplasm	√
	Tom70	45300	Mitochondria	Cytoplasm	√
**Endoplasmic Reticulum**	Gpi17	7520	ER	Cytoplasm	√
	Mmm1	2490	ER	Cytoplasm	X
	Pho88	61800	ER	Cytoplasm	√
	Sec63	17700	ER	Cytoplasm	√
	Yet1	7210	ER	Cytoplasm	√
**Vacuole**	Mam3	5890	Vac. Membrane	Cytoplasm	X
	Vph1	55700	Vac. Membrane	Cytoplasm	√
	Zrc1	8200	Vac. Membrane	Cytoplasm	√
**Lipid Droplets**	Faa4	31200	LD	Unknown	√
	Osw5	922	LD	Cytoplasm	√
**Plasma Membrane**	Ina1	4280	Cell Periphery	Cytoplasm	√
	Mep3	3820	Cell Periphery	Cytoplasm	√
	Pdr5	42000	Cell Periphery	Cytoplasm	X
	Yor1	3610	Cell Periphery	Cytoplasm	√
**Golgi**	Akr1	4070	Golgi	Cytoplasm	X
	Mnn9	1630	Golgi	Cytoplasm	X
	Rbd2	7850	Golgi	Cytoplasm	X

Supplementary Table 2 – Genes whose overexpression led to expansion of the split PerMit reporter signal.

Gene Name	ORF Name	mCherry Localization
ALY1	*YKR021W*	Cytosol
ANT1	*YPR128C*	Punctate, Mitochondria
ATG36	*YJL185C*	Punctate
BPH1	*YCR032W*	Vacuole membrane
CAF4	*YKR036C*	Mitochondria
COS6	*YGR295C*	Vacuole
DJP1	*YIR004W*	Cell periphery, ER, Bud
ELO1	*YJL196C*	ER
FIS1	*YIL065c*	Mitochondria
FZO1	*YBR179c*	Mitochondria
GEM1	*YAL048C*	Mitochondria
IDI1	*YPL117C*	Cytosol
IFA38	*YBR159W*	Punctate
INP1	*YMR204C*	Punctate
INP2	*YMR163C*	Punctate
LDB19	*YOR322C*	Cytosol, punctate
LYS1	*YIR034C*	Punctate
MDM10	*YAL010C*	Mitochondria
MIM1	*YOL026c*	Mitochondria
PCS60	*YBR222C*	Punctate
PEX1	*YKL197C*	Cytosol
PEX11	*YOL147C*	Punctate
PEX12	*YMR026C*	Punctate
PEX13	*YLR191W*	Punctate
PEX15	*YOL044W*	Punctate
PEX2	*YJL210W*	Punctate
PEX27	*YOR193W*	Punctate
PEX3	*YDR329C*	Punctate
PEX34	*YCL056C*	Punctate
PEX7	*YDR142C*	Punctate
PHO88	*YBR106W*	ER
RVS167	*YDR388W*	Cytosol, Punctate
SCT1	*YBL011W*	ER
SLM4	*YBR077C*	Vacuole membrane
SPS19	*YNL202W*	Punctate
STE23	*YLR389C*	Punctate, Mitochondria
STR3	*YGL184C*	Punctate
TES1	*YJR019C*	Punctate
VPS1	*YKR001C*	Cytosol, Punctate
VPS24	*YKL041W*	Cytosol
YDJ1	*YNL064C*	Cytosol
YPT1	*YFL038C*	Punctate
	YBL010C	Punctate

Supplementary Table 3. Plasmids used in this study.

MS - Maya Schuldiner lab plasmids; MC - Mickael Cohen lab plasmids; ME - Mafalda Escobar lab plasmids.

Plasmid number	Plasmid name	Description	Bacterial selection	Plasmid set	Plasmid use	Reference
MS47	pFA6a-KAN	Plasmid usefull as template for PCR for performing gene deletions, introducing the KAN selection marker.	Amp	pFA6	Gene deletion	(Longtine et al., 1998)[1]
MS49	pFA6a-NAT	Plasmid usefull as template for PCR for performing gene deletions, introducing the NAT selection marker.	Amp	pFA6	Gene deletion	(Goldstein et al., 1999)[2]
MS107	URA::TEF2pr-mCherry	Plasmid usefull as template for PCR in order to introduce a fluorescent mCherry reporter. May also be used for gene deletions or N' terminall tagging of proteins.	Amp	pFA6	Tag fusion and protein over expression	(Breslow et al ,2008)[3]
MS108	pFA6-Hygro	pFA6 based plasmid for KOs with Hygromycin resistance cassette	Amp	pFA6	Gene deletion	(Goldstein et al., 1999)[2]
MS446	pFA6a-VC-His3MX6	Plasmid for C terminal tagging of ORF with split YFP (C terminal fragment). Sequence in GenBank under Accession No: EF210803	Amp	pFA6	Tag fusion	(Sung and Huh, 2007)[4]
MS445	pFA6a-VN-kanMX6	Plasmid for C terminal tagging of ORF with split YFP (N terminal fragment). Sequence in GenBank under Accession No: EF210808	Amp	pFA6	Tag fusion	(Sung and Huh, 2007)[4]
MS457	pFA6a-kanMX6-PCET1-VN	Plasmid for constitutive expression of N terminally tagged ORF with split YFP (N terminal fragment). Sequence in GenBank under Accession No: EF521887	Amp	pFA6	Tag fusion	(Sung and Huh, 2007)[4]
MS451	pFA6a-kanMX6-PGAL1-VN	Plasmid for inducible expression of N terminally tagged ORF with split YFP (N terminal fragment). Sequence in GenBank under Accession No: EF210812	Amp	pFA6	Tag fusion	(Sung and Huh, 2007)[4]
MS686	pFA6a-VC-Hygro	Plasmid for C terminal tagging of ORF with split YFP (C terminal fragment). Sequence in GenBank under Accession No: EF210803. Modified from (Sung and Huh, 2007) by Maria Bohnert, Schuldiner Lab (introduction of Hygro)	Amp	pFA6	Tag fusion	This Study
MS700	pFA6a-NAT-PCET1-VN	Plasmid for constitutive expression of N terminally tagged ORF with split YFP (N terminal fragment). . Sequence in GenBank under Accession No: EF521883. Modifierd based on plasmid 455 for tagging with VN at the N with NAT instead of HIS selection. Modified from (Sung and Huh, 2007) by Yoav Peleg, Wiezmann instetute.	Amp	pFA6	Tag fusion	This Study
MS729	pAG25-IFP-F1	split far red reporter IFP F1 NAT	Amp	pFA6	Tag fusion	(Tchekanda et al, 2014)[5]
MS730	pAG32-IFP-F2	split far red reporter IFP F2 Hygro	Amp	pFA6	Tag fusion	(Tchekanda et al, 2014)[5]
MS21	pFA6a-GFP(S65T)-HIS3MX6	plasmid for making C'terminal GFP fusion proteins by PCR .	Amp	pFA6	Tag fusion	(Longtine et al., 1998)[1]
MS22	pFA6a-GFP(S65T)-KanMX6	plasmid for making C'terminal GFP fusion proteins by PCR .	Amp	pFA6	Tag fusion	(Longtine et al., 1998)[1]
MS179	TEF2-GFP-ADH1	Plasmid For N terminally tagging any protein with GFP or KO a protein and replacing it with GFP	Amp	pFA6	Tag fusion	David Breslow
MS278	pFa6a-mCherry-Nat	Plasmid for making C terminal mCherry fusion proteins. Made by cutting pFA6a-Cherry-His3MX and pFa6a-NAT with Asc I/Sac, ligation of NAT fragment into the same site in pFa6a-Cherry<br>Clone 1. Verified by restriction using Stu I / Sph I.	Amp	pFA6	Tag fusion	Tomer Ravid
MS371	pADHpr mtBFP426	BFP targeted to mitochondria	Amp	2micron	Expression plasmid	Christian Ungermann
MS618	pLC06:pRS416_PEX5pr_CFP-SNAP-SKL	Plasmid to express CFP with a PTS1 signal	Amp	pRS416	Expression plasmid	Ralf Erdmann
MS616	pAR2::GFP-SKL	pAR2::GFP-SKL	Amp	CEN	Expression plasmid	Ralf Erdmann
MC374	JMG435	pRS316-mRFP-SKL	Amp	CEN	Expression plasmid	(Fagarasanu, et al 2009)[6]
ME8	pRS316	Empty vector pRS316, cen, Ura3	Amp	pRS	Cloning	(Sikorski and Hieter, 1989)[7]
ME10	HA-Fzo1 on pRS316	HA-Fzo1 on pRS316, cen, Ura3	Amp	pRS	Expression of HA-Fzo1	(Escobar-Henriques et al., 2006)[8]

Supplementary Table 4. Yeast strains used in this study.

MS - Maya Schuldiner lab yeast strains ; MC - Mickael Cohen lab yeast strains

Strain Number	Strain Name	Genotype	Mating Type	Comments
MS2714	Split NVJ	his3delta1 leu2delta0 lys2+/lys+ met15delta0 ura3delta0 can1Δ::STE2pr-sp HIS5 lyp1Δ::STE3pr-LEU2; Zrc1-VC-His; Sec63-VN-Kan	alpha	
MS2717	Split MAM	his3delta1 leu2delta0 lys2+/lys+ met15delta0 ura3delta0 can1Δ::STE2pr-sp HIS5 lyp1Δ::STE3pr-LEU2; Tom20-VC-His; Sec63-VN-Kan	alpha	
MS2814	Split vCLAMP	his3delta1 leu2delta0 lys2+/lys+ met15delta0 ura3delta0 can1Δ::STE2pr-sp HIS5 lyp1Δ::STE3pr-LEU2; Tom20-VC-His; Zrc1-VN-Kan	alpha	
MS2815	Split NVJ + Nvj1-mCherry	his3delta1 leu2delta0 lys2+/lys+ met15delta0 ura3delta0 can1Δ::STE2pr-sp HIS5 lyp1Δ::STE3pr-LEU2; Zrc1-VC-His; Sec63-VN-Kan; Nvj1-mCherry-Nat	alpha	
MS2777	Split MAM + Mdm34-mCherry	his3delta1 leu2delta0 lys2+/lys+ met15delta0 ura3delta0 can1Δ::STE2pr-sp HIS5 lyp1Δ::STE3pr-LEU2; Tom20-VC-His; Sec63-VN-Kan; Mdm34-mCherry-Nat	alpha	
MS2819	Split NVJ + mCherry-Mdm1	his3delta1 leu2delta0 lys2+/lys+ met15delta0 ura3delta0 can1Δ::STE2pr-sp HIS5 lyp1Δ::STE3pr-LEU2; Zrc1-VC-His; Sec63-VN-Kan; Tef2-Ura-mCherry-Mdm1	alpha	
MS2774	Split NVJ + *Δvac8*	his3delta1 leu2delta0 lys2+/lys+ met15delta0 ura3delta0 can1Δ::STE2pr-sp HIS5 lyp1Δ::STE3pr-LEU2; Zrc1-VC-His; Sec63-VN-Kan; Δvac8:Nat	alpha	
MS2816	Split NVJ + *Δnvj1*	his3delta1 leu2delta0 lys2+/lys+ met15delta0 ura3delta0 can1Δ::STE2pr-sp HIS5 lyp1Δ::STE3pr-LEU2; Zrc1-VC-His; Sec63-VN-Kan; Δnvj1:Nat	alpha	
MS2817	Split MAM + Δ*mdm34*	his3delta1 leu2delta0 lys2+/lys+ met15delta0 ura3delta0 can1Δ::STE2pr-sp HIS5 lyp1Δ::STE3pr-LEU2; Tom20-VC-His; Sec63-VN-Kan; Δmdm34::Nat	alpha	
MS2818	Split MAM + Δ*mmm1*	his3delta1 leu2delta0 lys2+/lys+ met15delta0 ura3delta0 can1Δ::STE2pr-sp HIS5 lyp1Δ::STE3pr-LEU2; Tom20-VC-His; Sec63-VN-Kan; Δmmm1::Nat	alpha	
MS3871	Split ER-PM on SEY6210.1	leu2-3,112 ura3-52 his3-Δ 200 trp1-Δ901 lys2-801 suc2-Δ9 ; Sec63-VC-Hygro ; Nat-PCET-VN-Pmp3	a	SEY6210.1
MS3880	Δ6 tether + Split ER-PM	leu2-3,112 ura3-52 his3-Δ 200 trp1-Δ901 lys2-801 suc2-Δ9; ist2Δ::HISMX6 scs2Δ::TRP1 scs22Δ::HISMX6 tcb1Δ::KANMX6 tcb2Δ::KANMX6 tcb3Δ::HISMX6 ; Sec63-VC-Hygro ; Nat-PCET-VN-Pmp3	a	SEY6210.1
MS2778	Split MAM + mCherry-Lam6	his3delta1 leu2delta0 lys2+/lys+ met15delta0 ura3delta0 can1Δ::STE2pr-sp HIS5 lyp1Δ::STE3pr-LEU2; Tom20-VC-His; Sec63-VN-Kan; Tef2-Ura-mCherry-Lam6	alpha	
MS2820	Split vCLAMP + mCherry-Vam6	his3delta1 leu2delta0 lys2+/lys+ met15delta0 ura3delta0 can1Δ::STE2pr-sp HIS5 lyp1Δ::STE3pr-LEU2; Tom20-VC-His; Zrc1-VN-Kan; Tef2-Ura-mCherry-Vam6	alpha	
MS2881	Split PM-Mito	his3Δ1 leu2Δ0 met15Δ0 ura3Δ0; Ina1-VC-His; Tom70-VN-Kan	a	
MS2883	Split Vac-PM	his3Δ1 leu2Δ0 met15Δ0 ura3Δ0; Ina1-VC-His; Zrc1-VN-Kan	a	
MS3441	Split ER-PM	his3Δ1 leu2Δ0 met15Δ0 ura3Δ0; Ina1-VC-His; Pho88-VN-Kan	a	
MS2715	Split LD-Mito	his3delta1 leu2delta0 lys2+/lys+ met15delta0 ura3delta0 can1Δ::STE2pr-sp HIS5 lyp1Δ::STE3pr-LEU2; Osw5-VC-His; Tom70-VN-Kan	alpha	
MS2882	Split LD-PM	his3Δ1 leu2Δ0 met15Δ0 ura3Δ0; Ina1-VC-His; Osw5-VN-Kan	a	
MS2716	Split LD-Vac	his3delta1 leu2delta0 lys2+/lys+ met15delta0 ura3delta0 can1Δ::STE2pr-sp HIS5 lyp1Δ::STE3pr-LEU2; Osw5-VC-His; Zrc1-VN-Kan	alpha	
MS2885	Split LD-ER	his3Δ1 leu2Δ0 met15Δ0 ura3Δ0; Osw5-VC-His; Pho88-VN-Kan	a	
MS2658	Split PerMit 1	his3delta1 leu2delta0 lys2+/lys+ met15delta0 ura3delta0 can1Δ::STE2pr-sp HIS5 lyp1Δ::STE3pr-LEU2; Tom20-VC-His; Pex11-VN-Kan	alpha	
MS3442	Split Pex-PM	his3delta1 leu2delta0 lys2+/lys+ met15delta0 ura3delta0 can1Δ::STE2pr-sp HIS5 lyp1Δ::STE3pr-LEU2; Mep3-VC-His; Pex3-VN-Kan	alpha	
MS3443	Split Pex-Vac	his3delta1 leu2delta0 lys2+/lys+ met15delta0 ura3delta0 can1Δ::STE2pr-sp HIS5 lyp1Δ::STE3pr-LEU2; Zrc1-VC-His; Pex25-VN-Kan	alpha	
MS3444	Split Pex-ER	his3delta1 leu2delta0 lys2+/lys+ met15delta0 ura3delta0 can1Δ::STE2pr-sp HIS5 lyp1Δ::STE3pr-LEU2; Sec63-VC-His; Pex3-VN-Kan	alpha	
MS2098	Split Pex-LD	his3delta1 leu2delta0 lys2+/lys+ met15delta0 ura3delta0 can1Δ::STE2pr-sp HIS5 lyp1Δ::STE3pr-LEU2; Osw5-VC-His; Pex3-VN-Kan	alpha	
MS2097	Split PerMit 2	his3delta1 leu2delta0 lys2+/lys+ met15delta0 ura3delta0 can1Δ::STE2pr-sp HIS5 lyp1Δ::STE3pr-LEU2; Tom70-VC-His; Pex3-VN-Kan	alpha	
MS3445	Split PerMit 3	his3delta1 leu2delta0 lys2+/lys+ met15delta0 ura3delta0 can1Δ::STE2pr-sp HIS5 lyp1Δ::STE3pr-LEU2; Tom70-VC-His; Pex11-VN-Kan	alpha	
MS3446	Split PerMit 4	his3delta1 leu2delta0 lys2+/lys+ met15delta0 ura3delta0 can1Δ::STE2pr-sp HIS5 lyp1Δ::STE3pr-LEU2; Tom20-VC-His; Pex25-VN-Kan	alpha	
MS2094	Split PerMit 5	his3delta1 leu2delta0 lys2+/lys+ met15delta0 ura3delta0 can1Δ::STE2pr-sp HIS5 lyp1Δ::STE3pr-LEU2; Tom70-VC-His; Pex25-VN-Kan	alpha	
MS2100	Split PerMit 6	his3delta1 leu2delta0 lys2+/lys+ met15delta0 ura3delta0 can1Δ::STE2pr-sp HIS5 lyp1Δ::STE3pr-LEU2; Tom70-VC-His; Pex25-VN-Nat	alpha	
MS2663	Split PerMit 5 + Tom20-mCherry	his3delta1 leu2delta0 lys2+/lys+ met15delta0 ura3delta0 can1Δ::STE2pr-sp HIS5 lyp1Δ::STE3pr-LEU2; Tom70-VC-His; Pex25-VN-Kan; Tom20-mCherry-Nat	alpha	
MS2660	Split PerMit 5 + Pda1-mCherry	his3delta1 leu2delta0 lys2+/lys+ met15delta0 ura3delta0 can1Δ::STE2pr-sp HIS5 lyp1Δ::STE3pr-LEU2; Tom70-VC-His; Pex25-VN-Kan; Pda1-mCherry-Nat	alpha	
MS2659	Split PerMit 5 + Mdm34-mCherry	his3delta1 leu2delta0 lys2+/lys+ met15delta0 ura3delta0 can1Δ::STE2pr-sp HIS5 lyp1Δ::STE3pr-LEU2; Tom70-VC-His; Pex25-VN-Nat; Mdm34-mCherry-Kan	alpha	
MS3447	Split PerMit 5 + Mdm10 OE	his3delta1 leu2delta0 lys2+/lys+ met15delta0 ura3delta0 can1Δ::STE2pr-sp HIS5 lyp1Δ::STE3pr-LEU2; can1Δ::GAL1pr-SceI::STE2pr-SpHIS5 lyp1Δ::STE3pr-LEU2 ; NAT::TEF2pr-mCherry-Mdm10; Tom70-VC-His; Pex25-VN-Kan	alpha	
MS3448	Split PerMit 5+ Pcs60 OE	his3delta1 leu2delta0 lys2+/lys+ met15delta0 ura3delta0 can1Δ::STE2pr-sp HIS5 lyp1Δ::STE3pr-LEU2; can1Δ::GAL1pr-SceI::STE2pr-SpHIS5 lyp1Δ::STE3pr-LEU2 ; NAT::TEF2pr-mCherry-PCS60; Tom70-VC-His; Pex25-VN-Kan	alpha	
MS3449	Split PerMit 5+ Gem1 OE	his3delta1 leu2delta0 lys2+/lys+ met15delta0 ura3delta0 can1Δ::STE2pr-sp HIS5 lyp1Δ::STE3pr-LEU2; can1Δ::GAL1pr-SceI::STE2pr-SpHIS5 lyp1Δ::STE3pr-LEU2 ; NAT::TEF2pr-mCherry-Gem1; Tom70-VC-His; Pex25-VN-Kan	alpha	
MS3450	Split PerMit 5+ Caf4 OE	his3delta1 leu2delta0 lys2+/lys+ met15delta0 ura3delta0 can1Δ::STE2pr-sp HIS5 lyp1Δ::STE3pr-LEU2; can1Δ::GAL1pr-SceI::STE2pr-SpHIS5 lyp1Δ::STE3pr-LEU2 ; NAT::TEF2pr-mCherry-Caf4; Tom70-VC-His; Pex25-VN-Kan	alpha	
MS3451	Split PerMit 5 + Pex34 OE	his3delta1 leu2delta0 lys2+/lys+ met15delta0 ura3delta0 can1Δ::STE2pr-sp HIS5 lyp1Δ::STE3pr-LEU2; can1Δ::GAL1pr-SceI::STE2pr-SpHIS5 lyp1Δ::STE3pr-LEU2 ; NAT::TEF2pr-mCherry-Pex34; Tom70-VC-His; Pex25-VN-Kan	alpha	

MS3452	Split PerMit 5 + Fis1 OE	his3delta1 leu2delta0 lys2+/lys+ met15delta0 ura3delta0 can1Δ::STE2pr-sp HIS5 lyp1Δ::STE3pr-LEU2; can1Δ::GAL1pr-SceI::STE2pr-SpHIS5 lyp1Δ::STE3pr-LEU2 ; NAT::TEF2pr-mCherry-Fis1; Tom70-VC-His; Pex25-VN-Kan	alpha	
MS3453	Split PerMit 5 + Pex11 OE	his3delta1 leu2delta0 lys2+/lys+ met15delta0 ura3delta0 can1Δ::STE2pr-sp HIS5 lyp1Δ::STE3pr-LEU2; can1Δ::GAL1pr-SceI::STE2pr-SpHIS5 lyp1Δ::STE3pr-LEU2 ; NAT::TEF2pr-mCherry-Pex11; Tom70-VC-His; Pex25-VN-Kan	alpha	
MS3454	Split PerMit 5 + Fzo1 OE	his3delta1 leu2delta0 lys2+/lys+ met15delta0 ura3delta0 can1Δ::STE2pr-sp HIS5 lyp1Δ::STE3pr-LEU2; can1Δ::GAL1pr-SceI::STE2pr-SpHIS5 lyp1Δ::STE3pr-LEU2 ; NAT::TEF2pr-mCherry-Fzo1; Tom70-VC-His; Pex25-VN-Kan	alpha	
MS3455	Split PerMit 5 + Ste23 OE	his3delta1 leu2delta0 lys2+/lys+ met15delta0 ura3delta0 can1Δ::STE2pr-sp HIS5 lyp1Δ::STE3pr-LEU2; can1Δ::GAL1pr-SceI::STE2pr-SpHIS5 lyp1Δ::STE3pr-LEU2 ; NAT::TEF2pr-mCherry-Ste23; Tom70-VC-His; Pex25-VN-Kan	alpha	
MS3456	Split PerMit 5+ Inp2 OE	his3delta1 leu2delta0 lys2+/lys+ met15delta0 ura3delta0 can1Δ::STE2pr-sp HIS5 lyp1Δ::STE3pr-LEU2; can1Δ::GAL1pr-SceI::STE2pr-SpHIS5 lyp1Δ::STE3pr-LEU2 ; NAT::TEF2pr-mCherry-Inp2; Tom70-VC-His; Pex25-VN-Kan	alpha	
MS3457	Split PerMit 5 + Ant1 OE	his3delta1 leu2delta0 lys2+/lys+ met15delta0 ura3delta0 can1Δ::STE2pr-sp HIS5 lyp1Δ::STE3pr-LEU2; can1Δ::GAL1pr-SceI::STE2pr-SpHIS5 lyp1Δ::STE3pr-LEU2 ; NAT::TEF2pr-mCherry-Ant1; Tom70-VC-His; Pex25-VN-Kan	alpha	
MS3458	Split PerMit 5+ Inp1 OE	his3delta1 leu2delta0 lys2+/lys+ met15delta0 ura3delta0 can1Δ::STE2pr-sp HIS5 lyp1Δ::STE3pr-LEU2; can1Δ::GAL1pr-SceI::STE2pr-SpHIS5 lyp1Δ::STE3pr-LEU2 ; NAT::TEF2pr-mCherry-Inp1; Tom70-VC-His; Pex25-VN-Kan	alpha	
MS3459	Split PerMit 5 SGA Control	his3delta1 leu2delta0 lys2+/lys+ met15delta0 ura3delta0 can1Δ::STE2pr-sp HIS5 lyp1Δ::STE3pr-LEU2; can1Δ::GAL1pr-SceI::STE2pr-SpHIS5 lyp1Δ::STE3pr-LEU2 ; his3Δ1::NAT::TEF2pr-mCherry; Tom70-VC-His; Pex25-VN-Kan	alpha	
MS3296	Split PerMit 5 + Fzo1 OE	his3delta1 leu2delta0 lys2+/lys+ met15delta0 ura3delta0 can1Δ::STE2pr-sp HIS5 lyp1Δ::STE3pr-LEU2; Tom70-VC-His; Pex25-VN-Kan; Nat::Tef2-mCherry-Fzo1	alpha	
MS3298	Split PerMit 5 + N'-547 Truncated Fzo1 OE	his3delta1 leu2delta0 lys2+/lys+ met15delta0 ura3delta0 can1Δ::STE2pr-sp HIS5 lyp1Δ::STE3pr-LEU2; Tom70-VC-His; Pex25-VN-Kan; Nat::Tef2-mCherry-Fzo1-N'-547Truncated	alpha	
MS3740	Split NVJ Far Red	his3Δ1 leu2Δ0 met15Δ0 ura3Δ0 ;Zrc1-IFP-F2-Hygro;Sec63-IFP-F1-Nat	a	
MS3738	Split MAM Far Red	his3Δ1 leu2Δ0 met15Δ0 ura3Δ0 ; Sec63-IFP-F2-Hygro;Tom20-IFP-F1-Nat	a	
MS3974	Split ER-PM Far Red	his3Δ1 leu2Δ0 met15Δ0 ura3Δ0 ; Pho88-IFP-F2-Hygro;Ina1-IFP-F1-Nat	a	
MS3739	Split vCLAMP Far Red	his3Δ1 leu2Δ0 met15Δ0 ura3Δ0 ; Zrc1-IFP-F2-Hygro;Tom20-IFP-F1-Nat	a	
MS3741	Split Vac-PM Far Red	his3Δ1 leu2Δ0 met15Δ0 ura3Δ0 ; Ina1-IFP-F2-Hygro;Zrc1-IFP-F1-Nat	a	
MS3742	Split LD-PM Far Red	his3Δ1 leu2Δ0 met15Δ0 ura3Δ0 ; Ina1-IFP-F2-Hygro;Osw5-IFP-F1-Nat	a	
MS3744	Split Pex-Vac Far Red	his3Δ1 leu2Δ0 met15Δ0 ura3Δ0 ; Pex25-IFP-F2-Hygro;Zrc1-IFP-F1-Nat	a	
MS3743	Split Pex-PM Far Red	his3Δ1 leu2Δ0 met15Δ0 ura3Δ0 ; Ina1-IFP-F2-Hygro;Pex11-IFP-F1-Nat	a	
MS3735	Split PerMit Far Red	his3Δ1 leu2Δ0 met15Δ0 ura3Δ0 ; Tom70-IFP-F2-Hygro;Pex25-IFP-F1-Nat	a	
MS3857	Split MAM Far Red + Δ*mdm34*	his3Δ1 leu2Δ0 met15Δ0 ura3Δ0 ; Δmdm34:Kan; Sec63-IFP-F2-Hygro;Tom20-IFP-F1-Nat	a	
MS3858	Split MAM Far Red + Δmmm1	his3Δ1 leu2Δ0 met15Δ0 ura3Δ0 ; Δmmm1:Kan ; Sec63-IFP-F2-Hygro;Tom70-IFP-F1-Nat	a	
MS3865	Split PerMit Far Red + Pex3:GFP	his3Δ1 leu2Δ0 met15Δ0 ura3Δ0 ; Tom70-IFP-F2-Hygro;Pex25-IFP-F1-Nat ; Pex3:GFP-His	a	
MS3866	Split PerMit Far Red + Tom20:GFP	his3Δ1 leu2Δ0 met15Δ0 ura3Δ0 ; Tom70-IFP-F2-Hygro;Pex25-IFP-F1-Nat ; Tom20:GFP-His	a	
MS3867	Split PerMit Far Red + Pex34 OE	his3Δ1 leu2Δ0 met15Δ0 ura3Δ0 ; Tom70-IFP-F2-Hygro;Pex25-IFP-F1-Nat ; Tef2-GFP-Kan:Pex34	a	
MS3921	Split PerMit Far Red + Fzo1 OE	his3Δ1 leu2Δ0 met15Δ0 ura3Δ0 ; Tom70-IFP-F2-Hygro;Pex25-IFP-F1-Nat ; Tef2-GFP-Kan:Fzo1	a	
MS3301	Tom70-GFP + Fzo1 OE	his3Δ1 leu2Δ0 met15Δ0 ura3Δ0; Tom70-GFP-His; Nat::Tef2-mCherry-Fzo1	a	
MS3620	Tom70-GFP	his3Δ1 leu2Δ0 met15Δ0 ura3Δ0; Tom70-GFP-His	a	(Huh et al 2003)[9]. C' GFP library
MS3460	Pex3-VC + Cytosolic-VN	his3delta1 leu2delta0 lys2+/lys+ met15delta0 ura3delta0 can1Δ::STE2pr-sp HIS5 lyp1Δ::STE3pr-LEU2; Pex3-VC-His; Kan-PCET1-VN		
MS3461	Tom70-VC + Cytosolic-VN	his3Δ1 leu2Δ0 met15Δ0 ura3Δ0; Tom70-VC-His; Kan-PCET1-VN	a	
MS3462	Ina1-VC + Cytosolic-VN	his3Δ1 leu2Δ0 met15Δ0 ura3Δ0; Ina1-VC-His; Kan-PCET1-VN	a	
MS3463	Pho88-VC + Cytosolic-VN	his3delta1 leu2delta0 lys2+/lys+ met15delta0 ura3delta0 can1Δ::STE2pr-sp HIS5 lyp1Δ::STE3pr-LEU2; Pho88-VC-His; Kan-PCET1-VN		
MS3464	Osw5-VC + Cytosolic-VN	his3Δ1 leu2Δ0 met15Δ0 ura3Δ0; Osw5-VC-His; Kan-PCET1-VN	a	
MS3465	Zrc1-VC + Cytosolic-VN	his3Δ1 leu2Δ0 met15Δ0 ura3Δ0; Zrc1-VC-His; Kan-PCET1-VN	a	
MS3876	Inducible split PerMit + Mdm34:Cherry	his3Δ1 leu2Δ0 met15Δ0 ura3Δ0 ; Tom70-VC-His; Gal-VN-Kan-Pex25 ; Mdm34:Cherry:Nat	a	
MS3877	Inducible split PerMit + Pda1:Cherry	his3Δ1 leu2Δ0 met15Δ0 ura3Δ0 ; Tom70-VC-His; Gal-VN-Kan-Pex25 ; Pda1:Cherry:Nat	a	
MS2661	Split PerMit 5 + Pex11-mCherry	his3delta1 leu2delta0 lys2+/lys+ met15delta0 ura3delta0 can1Δ::STE2pr-sp HIS5 lyp1Δ::STE3pr-LEU2; Tom70-VC-His; Pex25-VN-Kan; Pex11-mCherry-Nat	alpha	
MS3466	Split PerMit 2 + Fzo1 OE	his3delta1 leu2delta0 lys2+/lys+ met15delta0 ura3delta0 can1Δ::STE2pr-sp HIS5 lyp1Δ::STE3pr-LEU2; Tom70-VC-His; Pex3-VN-Kan; Ura::Tef2-mCherry-Fzo1h	alpha	
MS3467	Split PerMit 1 + Fzo1 OE	his3delta1 leu2delta0 lys2+/lys+ met15delta0 ura3delta0 can1Δ::STE2pr-sp HIS5 lyp1Δ::STE3pr-LEU2; Tom20-VC-His; Pex11-VN-Kan; Ura::Tef2-mCherry-Fzo1	alpha	
MS3468	Split PerMit 5 + Pex34 OE	his3delta1 leu2delta0 lys2+/lys+ met15delta0 ura3delta0 can1Δ::STE2pr-sp HIS5 lyp1Δ::STE3pr-LEU2; Tom70-VC-His; Pex25-VN-Kan; Ura::Tef2-mCherry-Pex34	alpha	
MS3469	Split PerMit 2 + Pex34 OE	his3delta1 leu2delta0 lys2+/lys+ met15delta0 ura3delta0 can1Δ::STE2pr-sp HIS5 lyp1Δ::STE3pr-LEU2; Tom70-VC-His; Pex3-VN-Kan; Ura::Tef2-mCherry-Pex34	alpha	
MS3470	Split PerMit 1 + Pex34 OE	his3delta1 leu2delta0 lys2+/lys+ met15delta0 ura3delta0 can1Δ::STE2pr-sp HIS5 lyp1Δ::STE3pr-LEU2; Tom20-VC-His; Pex11-VN-Kan; Ura::Tef2-mCherry-Pex34	alpha	
MS3922	Split PerMit 5 + Δ*pex11*	his3delta1 leu2delta0 lys2+/lys+ met15delta0 ura3delta0 can1Δ::STE2pr-sp HIS5 lyp1Δ::STE3pr-LEU2; Tom70-VC-His; Pex25-VN-Kan; ΔPex11-Kan	alpha	

MS3923	Split PerMit 5 + Δ*fzo1*	his3delta1 leu2delta0 lys2+/lys+ met15delta0 ura3delta0 can1Δ::STE2pr-sp HIS5 lyp1Δ::STE3pr-LEU2; Tom70-VC-His; Pex25-VN-Kan; ΔFzo1-Kan	alpha	
MS3924	Split PerMit 5+ Δ*pex34*	his3delta1 leu2delta0 lys2+/lys+ met15delta0 ura3delta0 can1Δ::STE2pr-sp HIS5 lyp1Δ::STE3pr-LEU2; Tom70-VC-His; Pex25-VN-Kan; ΔPex34-Kan	alpha	
MS2561	Pex3-GFP	PEX3-GFP::HIS ; his3Δ1 leu2Δ0 met15Δ0 ura3Δ0	a	
MS3627	Pex3-GFP + Pex34 OE	his3delta1 leu2delta0 lys2+/lys+ met15delta0 ura3delta0 can1Δ::STE2pr-sp HIS5 lyp1Δ::STE3pr-LEU2; can1Δ::GAL1pr-SceI::STE2pr-SpHIS5 lyp1Δ::STE3pr-LEU2 ; NAT::TEF2pr-mCherry-Pex34; Pex3-GFP::His	alpha	
C' GFP library	Mdm34-GFP	his3Δ1 leu2Δ0 met15Δ0 ura3Δ0; Mdm34-GFP-His	a	(Huh et al 2003)[9]. C' GFP library
MS3473	Fzo1 OE + Mdm34-GFP	his3delta1 leu2delta0 lys2+/lys+ met15delta0 ura3delta0 can1Δ::STE2pr-sp HIS5 lyp1Δ::STE3pr-LEU2; can1Δ::GAL1pr-SceI::STE2pr-SpHIS5 lyp1Δ::STE3pr-LEU2 ; NAT::TEF2pr-mCherry-Fzo1 Mdm34-GFP-Kan	alpha	
MS3475	Pex34 OE + Mdm34-GFP	his3delta1 leu2delta0 lys2+/lys+ met15delta0 ura3delta0 can1Δ::STE2pr-sp HIS5 lyp1Δ::STE3pr-LEU2; can1Δ::GAL1pr-SceI::STE2pr-SpHIS5 lyp1Δ::STE3pr-LEU2 ; NAT::TEF2pr-mCherry-Pex34; Mdm34-GFP-Kan	alpha	
MS3477	Split MAM + Fzo1 OE	his3delta1 leu2delta0 lys2+/lys+ met15delta0 ura3delta0 can1Δ::STE2pr-sp HIS5 lyp1Δ::STE3pr-LEU2; Tom20-VC-His; Sec63-VN-Kan; Ura::Tef2-mCherry-Fzo1	alpha	
MS3478	Split MAM + Pex34 OE	his3delta1 leu2delta0 lys2+/lys+ met15delta0 ura3delta0 can1Δ::STE2pr-sp HIS5 lyp1Δ::STE3pr-LEU2; Tom20-VC-His; Sec63-VN-Kan; Ura::Tef2-mCherry-Pex34	alpha	
MS3976	Split Pex-PM + Pex34 OE	his3delta1 leu2delta0 lys2+/lys+ met15delta0 ura3delta0 can1Δ::STE2pr-sp HIS5 lyp1Δ::STE3pr-LEU2; Mep3-VC-His; Pex3-VN-Kan; Tef2-mCherry-URA-Pex34	alpha	
MS3977	Split Pex-Vac + Pex34 OE	his3delta1 leu2delta0 lys2+/lys+ met15delta0 ura3delta0 can1Δ::STE2pr-sp HIS5 lyp1Δ::STE3pr-LEU2; Zrc1-VC-His; Pex25-VN-Kan; Tef2-mCherry-URA-Pex34	alpha	
MS3978	Split Pex-ER + Pex34 OE	his3delta1 leu2delta0 lys2+/lys+ met15delta0 ura3delta0 can1Δ::STE2pr-sp HIS5 lyp1Δ::STE3pr-LEU2; Sec63-VC-His; Pex3-VN-Kan; Tef2-mCherry-URA-Pex34	alpha	
MS3979	Split Pex-LD + + Pex34 OE	his3delta1 leu2delta0 lys2+/lys+ met15delta0 ura3delta0 can1Δ::STE2pr-sp HIS5 lyp1Δ::STE3pr-LEU2; Osw5-VC-His; Pex3-VN-Kan; Tef2-mCherry-URA-Pex34	alpha	
MS3980	Split Pex-PM + Fzo1 OE	his3delta1 leu2delta0 lys2+/lys+ met15delta0 ura3delta0 can1Δ::STE2pr-sp HIS5 lyp1Δ::STE3pr-LEU2; Mep3-VC-His; Pex3-VN-Kan; Tef2-mCherry-URA-Fzo1	alpha	
MS3981	Split Pex-Vac + Fzo1 OE	his3delta1 leu2delta0 lys2+/lys+ met15delta0 ura3delta0 can1Δ::STE2pr-sp HIS5 lyp1Δ::STE3pr-LEU2; Zrc1-VC-His; Pex25-VN-Kan; Tef2-mCherry-URA-Fzo1	alpha	
MS3982	Split Pex-ER + Fzo1 OE	his3delta1 leu2delta0 lys2+/lys+ met15delta0 ura3delta0 can1Δ::STE2pr-sp HIS5 lyp1Δ::STE3pr-LEU2; Sec63-VC-His; Pex3-VN-Kan; Tef2-mCherry-URA-Fzo1	alpha	
MS3983	Split Pex-LD + Fzo1 OE	his3delta1 leu2delta0 lys2+/lys+ met15delta0 ura3delta0 can1Δ::STE2pr-sp HIS5 lyp1Δ::STE3pr-LEU2; Osw5-VC-His; Pex3-VN-Kan; Tef2-mCherry-URA-Fzo1	alpha	
MS3881	Pex25:GFP	his3Δ1 leu2Δ0 met15Δ0 ura3Δ0 ; Pex25:GFP:Kan	a	
MS3861	Tom70:GFP + Fzo1 OE	his3Δ1 leu2Δ0 met15Δ0 ura3Δ0 ; Tom70:GFP-His ;Tef2-mCherry-Ura:Fzo1	a	
MS3862	Tom70:GFP + Pex34 OE	his3Δ1 leu2Δ0 met15Δ0 ura3Δ0 ; Tom70:GFP-His ; Tef2-mCherry-Ura:Pex34	a	
MS3863	Pex25:GFP + Fzo1 OE	his3delta1 leu2delta0 lys2+/lys+ met15delta0 ura3delta0 can1Δ::STE2pr-sp HIS5 lyp1Δ::STE3pr-LEU2; can1Δ::GAL1pr-SceI::STE2pr-SpHIS5 lyp1Δ::STE3pr-LEU2 ;Tef2-mCherry-Nat:Fzo1 ; Pex25:GFP-Kan	alpha	
MS3864	Pex25:GFP + Pex34 OE	his3delta1 leu2delta0 lys2+/lys+ met15delta0 ura3delta0 can1Δ::STE2pr-sp HIS5 lyp1Δ::STE3pr-LEU2; can1Δ::GAL1pr-SceI::STE2pr-SpHIS5 lyp1Δ::STE3pr-LEU2 ;Tef2-mCherry-Nat:Pex34 ; Pex25:GFP-Kan	alpha	
MS3951	Pex34 OE + Δ*pex3*	his3delta1 leu2delta0 lys2+/lys+ met15delta0 ura3delta0 can1Δ::STE2pr-sp HIS5 lyp1Δ::STE3pr-LEU2; Mep3-VC-His; Pex3-VN-Kan; Tef2-mCherry-NAT-Pex34; Δpex3:KAN	a	
MS3859	Split PerMit + Fzo1 OE + ΔPex34	his3delta1 leu2delta0 lys2+/lys+ met15delta0 ura3delta0 can1Δ::STE2pr-sp HIS5 lyp1Δ::STE3pr-LEU2 ; NAT::TEF2pr-mCherry-Fzo1; Tom70-VC-His; Pex25-VN-Kan; ΔPex34:Hygro	a	
MS3952	Split PerMi + Pex34 OE + ΔFzo1	his3delta1 leu2delta0 lys2+/lys+ met15delta0 ura3delta0 can1Δ::STE2pr-sp HIS5 lyp1Δ::STE3pr-LEU2; Tom70-VC-His; Pex25-VN-Kan; Tef2:mCherry::Ura:Pex34; ΔFzo1-Kan	alpha	
MS3618	Split PerMit 5 + Pex34 OE + Δ*cit2*	his3delta1 leu2delta0 lys2+/lys+ met15delta0 ura3delta0 can1Δ::STE2pr-sp HIS5 lyp1Δ::STE3pr-LEU2; can1Δ::GAL1pr-SceI::STE2pr-SpHIS5 lyp1Δ::STE3pr-LEU2 ; NAT::TEF2pr-mCherry-Pex34; Tom70-VC-His; Pex25-VN-Kan; Δcit2-Hygro	alpha	
MS3619	Split PerMit 5 + Pex34 OE + Δ*cat2*	his3delta1 leu2delta0 lys2+/lys+ met15delta0 ura3delta0 can1Δ::STE2pr-sp HIS5 lyp1Δ::STE3pr-LEU2; can1Δ::GAL1pr-SceI::STE2pr-SpHIS5 lyp1Δ::STE3pr-LEU2 ; NAT::TEF2pr-mCherry-Pex34; Tom70-VC-His; Pex25-VN-Kan; Δcat2-Hygro	alpha	
MS3479	Δ*pex3*	his3Δ1 leu2Δ0 met15Δ0 ura3Δ0 ; Δpex3::Kan	a	
SWAT N' mCherry library	SWAT control	his3delta1 leu2delta0 lys2+/lys+ met15delta0 ura3delta0 can1Δ::STE2pr-sp HIS5 lyp1Δ::STE3pr-LEU2; can1Δ::GAL1pr-SceI::STE2pr-SpHIS5 lyp1Δ::STE3pr-LEU2 ; his3Δ1::NAT::TEF2pr-mCherry	alpha	(Yofe et al., 2016)[10]. SWAT N' mCherry library
MS83	BY4741	his3Δ1 leu2Δ0 met15Δ0 ura3Δ0	a	WT
MS721	SGA Query	his3delta1 leu2delta0 lys2+/lys+ met15delta0 ura3delta0; can1Δ::STE2pr-sp HIS5 lyp1Δ::STE3pr-LEU2	alpha	SGA Query strain
MS115	S288C control+Kan	his3Δ1 leu2Δ0 met15Δ0 ura3Δ0::KAN	a	
MS1658	Δ*mdm34*	his3Δ1 leu2Δ0 met15Δ0 ura3Δ0; Δmdm34::Kan	a	
MS1661	Δ*mmm1*	his3Δ1 leu2Δ0 met15Δ0 ura3Δ0 ; Δmmm1::Kan	a	
SWAT N' GFP library	GFP-Pex14	his3Δ1 leu2Δ0 met15Δ0 ura3Δ0; hphΔn::URA3::SpNOP1pr-sfGFP-Pex14	a	(Yofe et al., 2016)[10]. SWAT N' mCherry library
SWAT N' GFP library	GFP-Pex19	his3Δ1 leu2Δ0 met15Δ0 ura3Δ0; hphΔn::URA3::SpNOP1pr-sfGFP-Pex19	a	(Yofe et al., 2016)[10]. SWAT N' mCherry library
MC1423	W303-Δ*mdm30* + Fzo1-GFP + pRS316-mRFP-SKL	ura3-1 trp1-1 leu2-3,112 his3-11,15 can1-100 RAD5 ADE2 mdm30::KanMX FZO1-EGFP::HIS3MX6 + pRS316-mRFP-SKL	a	W303
MC1119	W303-Fzo1-13Myc	ura3-1 trp1-1 leu2-3,112 his3-11,15 can1-100 RAD5 ADE2 FZO1-13Myc::His3MX6	a	W303
MC1131	W303-Δ*mdm30* + Fzo1-13Myc	ura3-1 trp1-1 leu2-3,112 his3-11,15 can1-100 RAD5 ADE2 mdm30::KanMX FZO1-13Myc::His3MX6	alpha	W303

Supplementary References

1. Longtine, M. S. et al. Additional modules for versatile and economical PCR-based gene deletion and modification in Saccharomyces cerevisiae. Yeast 14, 953-961, doi:10.1002/(SICI)1097-0061(199807)14:10<953::AID-YEA293>3.0.CO;2-U (1998).
2. Goldstein, A. L. & McCusker, J. H. Three new dominant drug resistance cassettes for gene disruption in Saccharomyces cerevisiae. Yeast 15, 1541-1553, doi:10.1002/(SICI)1097-0061(199910)15:14<1541::AID-YEA476>3.0.CO;2-K (1999).
3. Breslow, D. K. et al. A comprehensive strategy enabling high-resolution functional analysis of the yeast genome. Nature methods 5, 711-718, doi:10.1038/nmeth.1234 (2008).
4. Sung, M. K. & Huh, W. K. Bimolecular fluorescence complementation analysis system for in vivo detection of protein-protein interaction in Saccharomyces cerevisiae. Yeast 24, 767-775, doi:10.1002/yea.1504 (2007).
5. Tchekanda, E., Sivanesan, D. & Michnick, S. W. An infrared reporter to detect spatiotemporal dynamics of protein-protein interactions. Nature methods 11, 641-644, doi:10.1038/nmeth.2934 (2014).
6. Fagarasanu, A. et al. Myosin-driven peroxisome partitioning in S. cerevisiae. The Journal of cell biology 186, 541-554, doi:10.1083/jcb.200904050 (2009).
7. Sikorski, R. S. & Hieter, P. A System of Shuttle Vectors and Yeast Host Strains Designed for Efficient Manipulation of DNA in Saccharomyces-Cerevisiae. Genetics 122, 19-27 (1989).
8. Escobar-Henriques, M., Westermann, B. & Langer, T. Regulation of mitochondrial fusion by the F-box protein Mdm30 involves proteasome-independent turnover of Fzo1. The Journal of cell biology 173, 645-650, doi:10.1083/jcb.200512079 (2006).
9. Huh, W. K. et al. Global analysis of protein localization in budding yeast. Nature 425, 686-691, doi:10.1038/nature02026 (2003).
10. Yofe, I. et al. One library to make them all: streamlining the creation of yeast libraries via a SWAp-Tag strategy. Nature methods 13, 371-378, doi:10.1038/nmeth.3795 (2016).
